# Supplementary material for: A RNA producing DNA hydrogel as a platform for a high performance RNA interference system
Source: Nat Commun. 2018 Oct 18;9:4331. doi: 10.1038/s41467-018-06864-0 (PMC6193956; doi:10.1038/s41467-018-06864-0)
Supplement: Supplementary file 1 — Supplementary Information [file 41467_2018_6864_MOESM1_ESM.pdf]

Supplementary Information

A RNA producing DNA hydrogel as a platform for a high performance RNA interference system

Song et. al.

Correspondence should be addressed to pospnk@mju.ac.kr

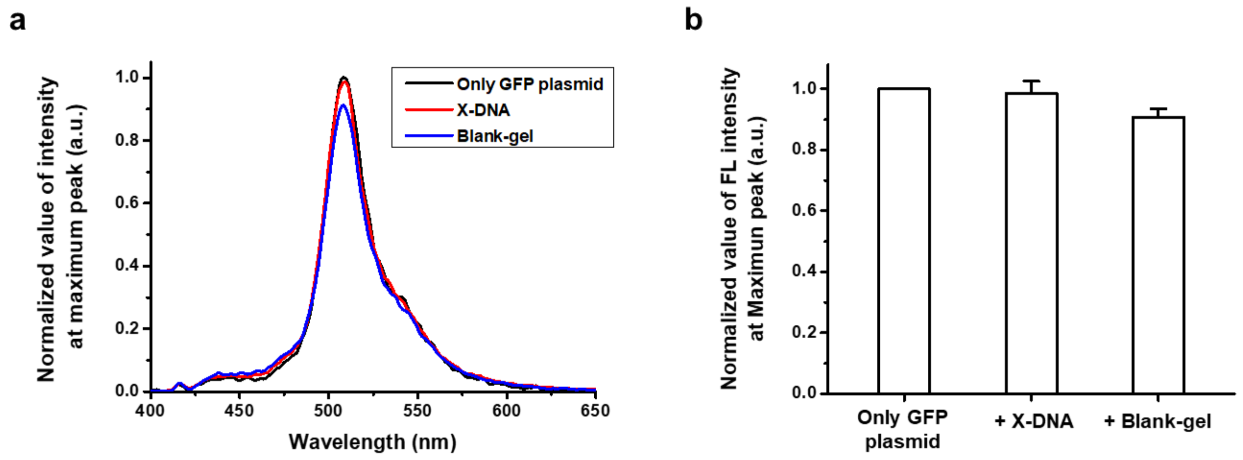

Supplementary Figure 1. The GFP expression test in X-DNA and Blank-gel (w/o I-plasmid) and samples. (a) The normalized FL spectra by three X-DNA and Blank-gel samples. (b) The relative value of the fluorescence intensity by each sample. Error bars refer to standard deviations from three replicates.

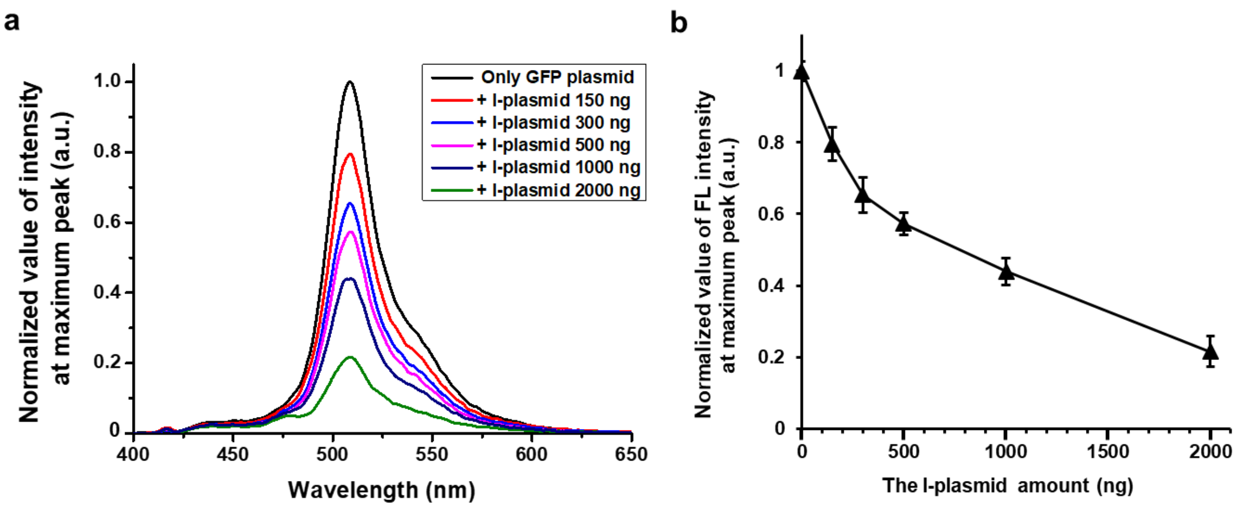

Supplementary Figure 2. GFP expression with increased I-plasmid amount. (a) The normalized FL spectra, (b) the ratio of peak intensity of expressed GFP with increased amount of I-plasmid (0, 150, 300, 500, 1000, and 2000 ng). Error bars refer to standard deviations from three replicates.

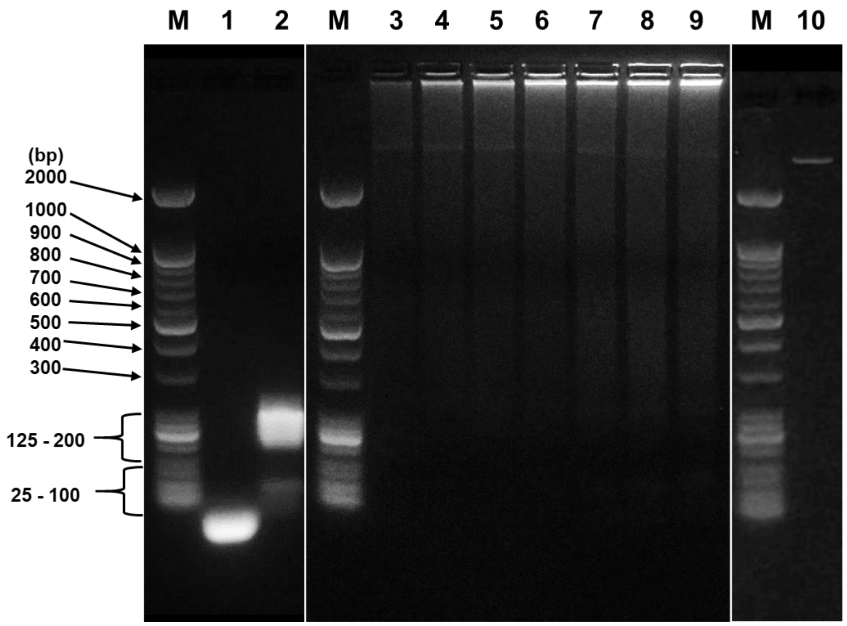

Supplementary Figure 3. Agarose gel electrophoresis image of the I-gel in various conditions. Note: DNA was resolved in 2% agarose gel stained with 1x Gel-red. Samples on gel are lane M: DNA marker (25~2,000 bps); lane 1: 36 base single strand DNA (one element composing of X-DNA); lane 2: 3 sticky-ends X-DNA; lane 3~9: 1000, 1500, 2000, 3000, 4000, 5000, 6000 X-DNA and I-plasmid ratio of nano-scale I-gel; lane 10: I-plasmid.

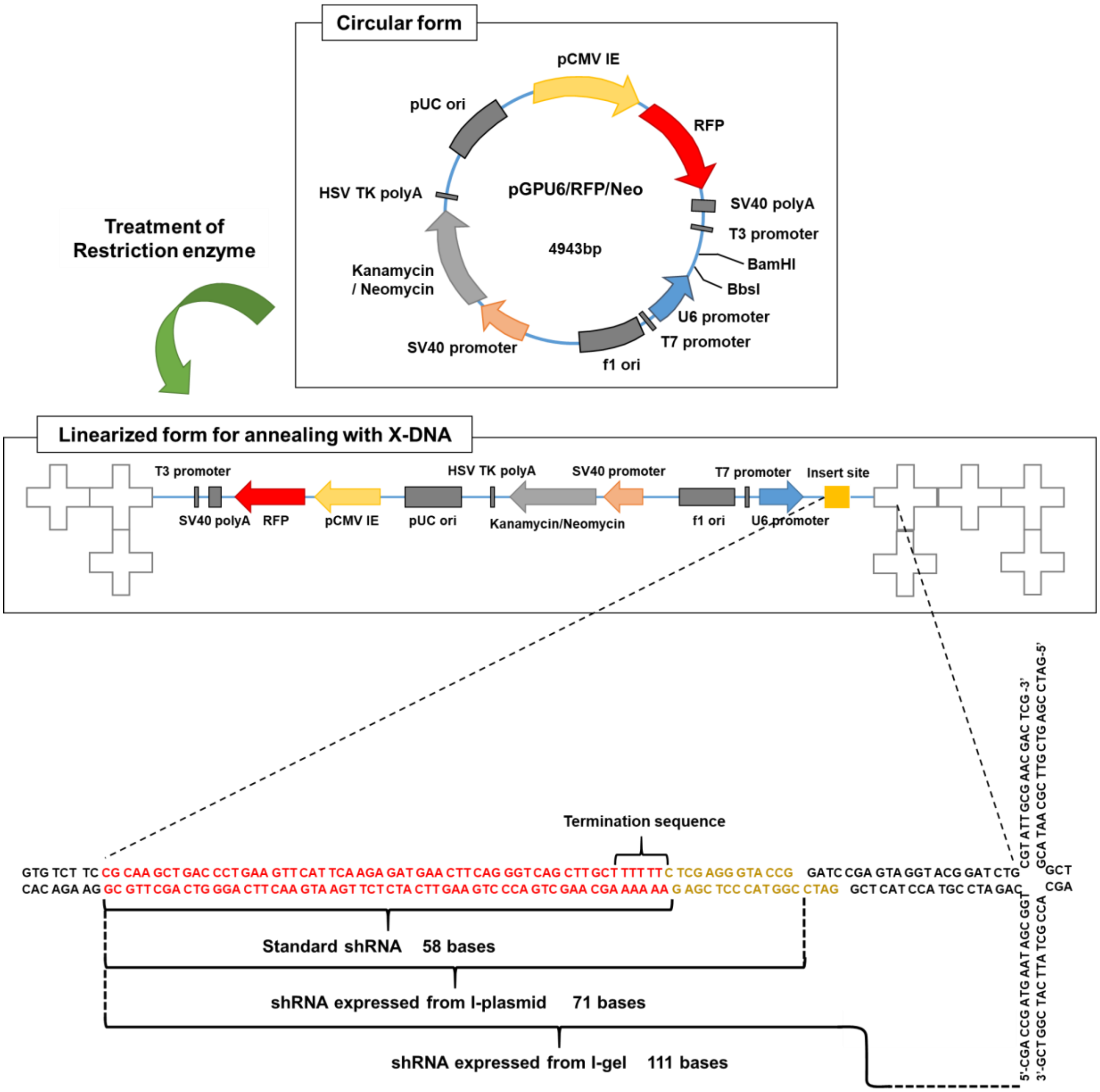

Supplementary Figure 4. The shRNA expression plasmid (I-plasmid) map and expected shRNA transcription: The designed sequence was inserted the site between BbsI and BamHI. This plasmid maxi-prepped for amplification and linearized by BamHI restriction enzyme. The restriction site is complementary and palindromic sequence with each X-DNA and plasmid itself. The X-DNAs and linearized plasmids can be linked by their sticky-ends. The I-gel can be synthesized with covalently conjugation of sticky-end through ligase enzyme reaction.

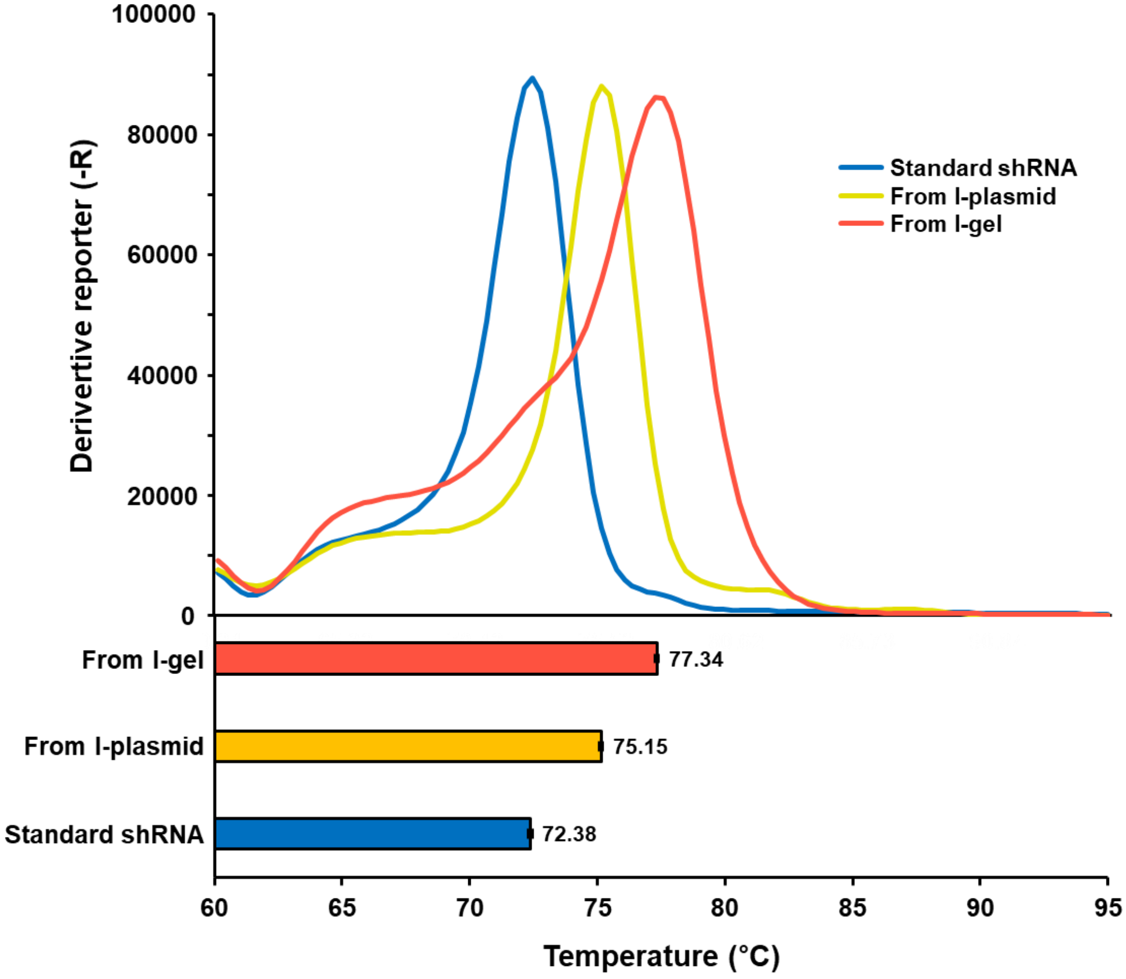

Supplementary Figure 5. The measured melting points of RT-PCR products. Blue: from standard shRNA, yellow: from free I-plasmid, and red: from I-gel. Error bars refer to standard deviations from three replicates.

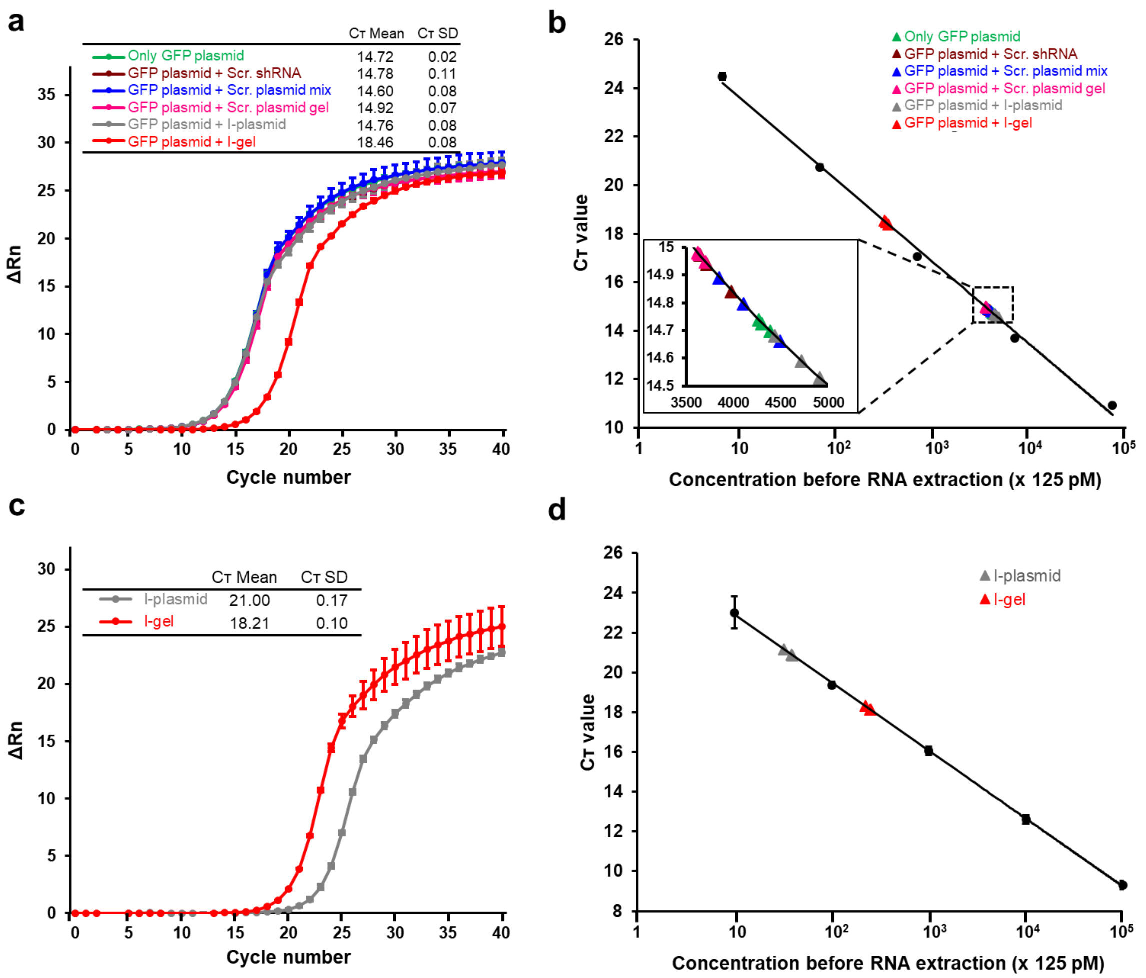

Supplementary Figure 6. CT and standard curves. qPCR curves and extracted CT values of (a) GFP mRNA and (c) shRNA. Standard curves for (b) GFP mRNA and (d) shRNA. Inset represents zoomed up image of dashed box. Error bars refer to standard deviations from three replicates.

Supplementary Table 1. RNA recovery rates and GFP mRNA concentration in each sample.

Sample	CT value (spike-in control)	Recovery Rate(%)	Conc. of GFP mRNA (nM)
ref cel-miR-39	19.37 ± 0.27	100	NA
Only GFP plasmid	19.58 ± 0.05	86.75 ± 2.56	622.0 ± 25.8
GFP plasmid + Scrambled shRNA	19.87 ± 0.04	71.73 ± 1.94	647.5 ± 43.0
GFP plasmid + Scrambled plasmid mix	19.57 ± 0.06	87.32 ± 3.50	593.6 ± 61.6
GFP plasmid + Scrambled I-gel	19.85 ± 0.05	72.99 ± 2.21	625.9 ± 25.0
GFP plasmid + I-plasmid	19.54 ± 0.06	89.25 ± 3.39	588.7 ± 47.6
GFP plasmid + I-gel	20.17 ± 0.04	59.04 ± 1.69	73.4 ± 5.2

The data show mean ± s.d., representative from 3 independent experiments. The EGFP mRNA amount was determined by multiplying each RNA recovery rate (%) and the value of RNA amount from the calibration curve (Supplementary Figure 6).

Supplementary Table 2. RNA recovery rates and shRNA concentration in each sample.

Sample	CT value (spike-in control)	Recovery Rate(%)	Conc. of shRNA (nM)
ref cel-miR-39	19.37 ± 0.27	100	NA
I-plasmid	20.92 ± 0.08	36.18 ± 1.97	12.7 ± 1.8
I-gel	21.09 ± 0.02	32.22 ± 0.47	91.9 ± 6.5

The data show mean ± s.d., representative from 3 independent experiments. The shRNA amount was determined by multiplying each RNA recovery rate (%) and the value of RNA amount from the calibration curve (Supplementary Figure 6).

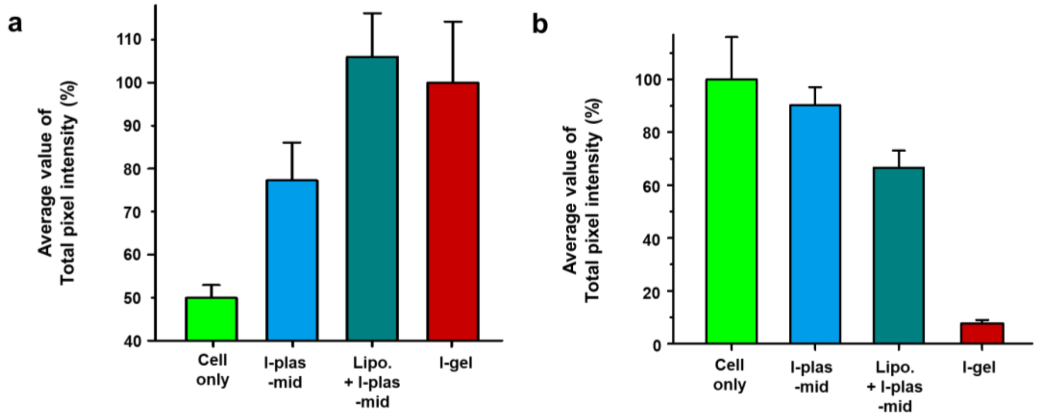

Supplementary Figure 7. The relative pixel intensities from (a) Cy5 and (b) GFP fluorescence images of non-treated MDCK-GFP cell (cell only), and treated cells with I-plasmid, I-plasmid complexed with Lipofectamine, and I-gel. Error bars refer to standard deviations from three replicates.

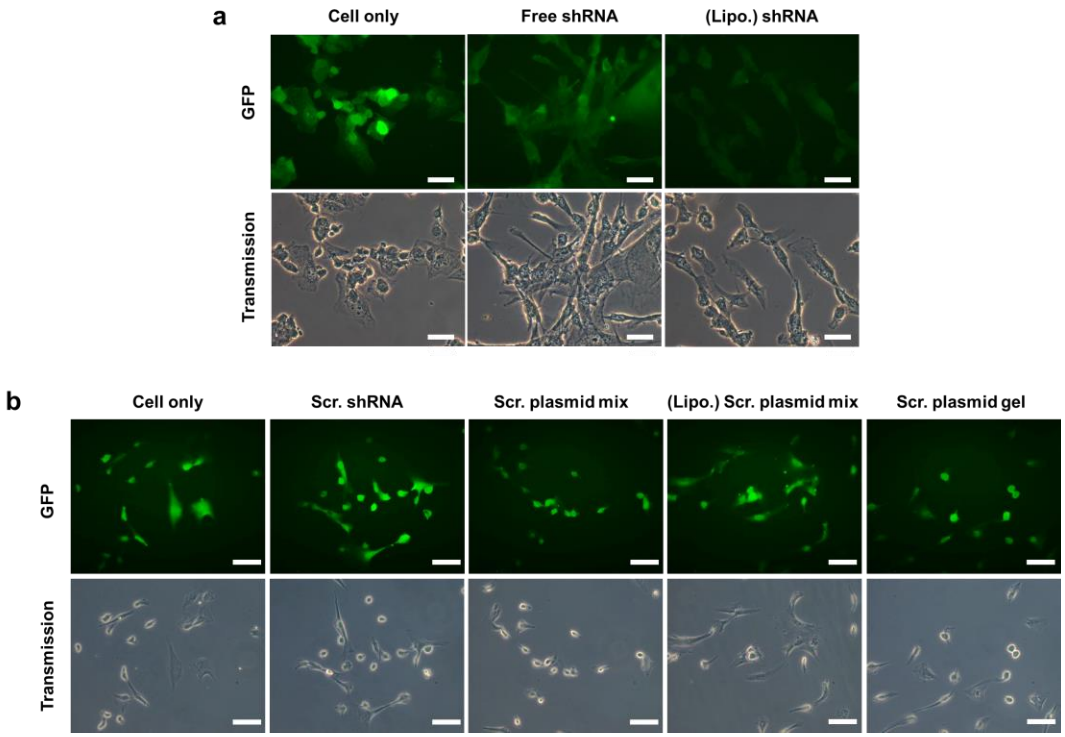

Supplementary Figure 8. Fluorescence images of MDCK GFP-expressing (MDCK-GFP) cells co-incubated with only shRNA and Lipofectamine-shRNA complex. (a) FL Images in condition of only shRNA and Lipofectamine-shRNA complex for positive control of RNA interference effect. (b) FL Images in condition of only scrambled shRNA, only scrambled plasmid mixture, Lipofectamine-plasmid mixture complex and scrambled plasmid gel for negative control of RNA interference effect. Scale bar: 50 μm.

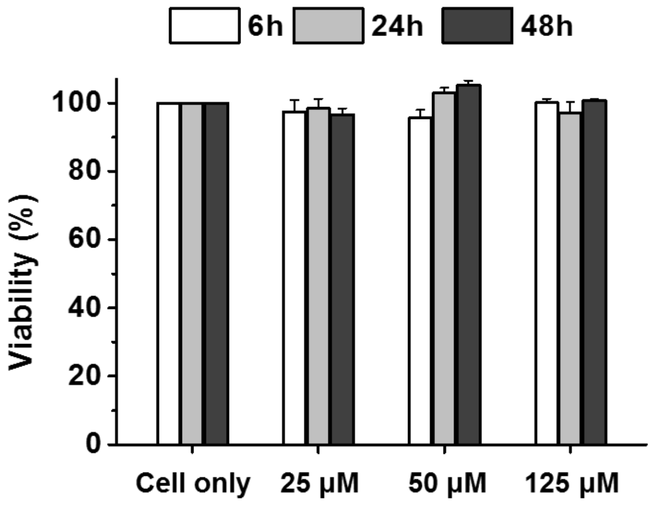

Supplementary Figure 9. Cytotoxicity test of I-gel. In various incubation times, cell viabilities of the MDCK-GFP cells co-incubated with each concentration of I-gel which is corresponding of X-DNA concentration. Error bars refer to standard deviations from three replicates.

Supplementary Table 3. One way ANOVA results of GFP mRNA level for multiple group analysis. (The case of that the cell only or I-gel condition was compared with other groups)

i) One way ANOVA table

ANOVA table	SS	DF	MS	F (DFn, DFd)	P value
Treatment (between columns)	3.1064	9	0.3452	F (9, 20) = 5.5477	P=0.0007
Residual (within columns)	1.2443	20	0.0622		
Total	4.3507	29			

Note: (SS: Sum of Squares, DF: Degree of Freedom, MS: Mean Square)

ii) Holm Bonferroni's multiple comparisons test table (Cell only vs)

Holm Bonferroni's multiple comparisons test	Mean Diff.	t	Holm P Value	Summary
Cell only vs. Scrambled shRNA	-0.1995	0.9797	>0.9999	ns
Cell only vs. Scrambled plasmid mixture	-0.1354	0.6647	>0.9999	ns
Cell only vs. Scrambled plasmid gel	0.0674	0.3308	>0.9999	ns
Cell only vs. Lipo+Scrambled plasmid mixture	0.1038	0.5096	>0.9999	ns
Cell only vs. I-plasmid	0.2085	1.024	>0.9999	ns
Cell only vs. Lipo+I-plasmid	0.3446	1.692	0.6368	ns
Cell only vs. shRNA	0.4663	2.290	0.2313	ns
Cell only vs. Lipo+shRNA	0.7582	3.723	0.0121	*
Cell only vs. I-gel	0.7537	3.701	0.0113	*

Note: (t: Bonferroni T-statistic)
*Asterisks indicate statistically significant differences between each group and Cell only.

iii) Holm Bonferroni's multiple comparisons test table (I-gel vs)

Holm Bonferroni's multiple comparisons test	Mean Diff.	t	Holm P Value	Summary
I-gel vs. Cell only	-0.7537	3.701	0.0099	*
I-gel vs. Scrambled shRNA	-0.9532	4.681	0.0013	**
I-gel vs. Scrambled plasmid mixture	-0.8891	4.365	0.0024	**
I-gel vs. Scrambled plasmid gel	-0.6863	3.370	0.0183	*
I-gel vs. Lipo+Scrambled plasmid mixture	-0.6499	3.191	0.0229	*
I-gel vs. I-plasmid	-0.5452	2.677	0.0580	ns
I-gel vs. Lipo+I-plasmid	-0.4091	2.009	0.1748	ns
I-gel vs. shRNA	-0.2874	1.411	0.3472	ns
I-gel vs. Lipo+shRNA	0.0045	0.0219	0.9828	ns

Note: (t: Bonferroni T-statistic)
*Asterisks indicate statistically significant differences between each group and I-gel.

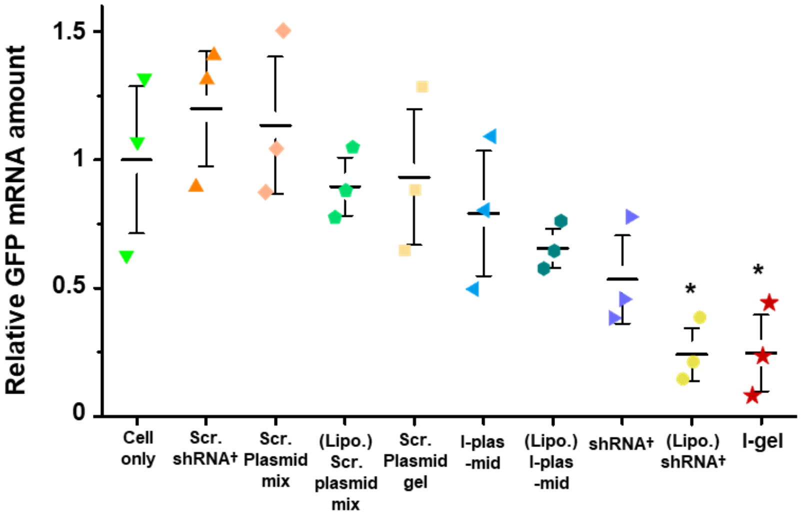

Supplementary Figure 10. Scattered plot type of the relative amounts of GFP mRNA. (†concentration of these conditions were 102 fold increased in consideration of the template to RNA transcription rate of the I-gel, *$P < 0.05$, analyzed by one-away ANOVA, followed by Holm-Bonferroni multiple comparisons post-test. Asterisks indicate statistically significant differences between each condition and Cell only)

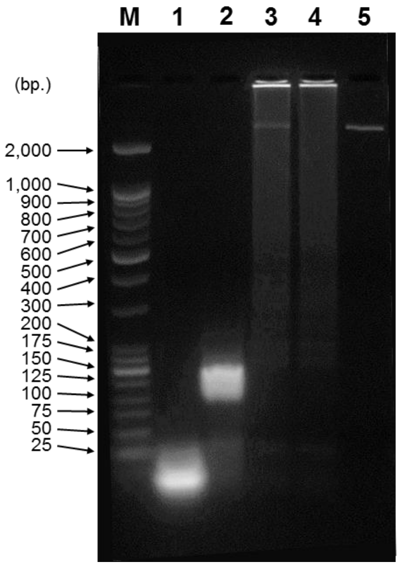

Supplementary Figure 11. Agarose gel electrophoresis image of the I-gel and its ingredients. Note: DNA was resolved on 2% agarose gel stained with 1x Gel-red. Samples on gel are lane M: DNA marker (25~2,000 bps); lane 1: 36 base single strand DNA (one element composing of X-DNA); lane 2: 3 sticky-ends X-DNA; lane 3: I-gel; lane 4: Blank-gel; lane 5: shRNA expressing plasmid (I-plasmid).

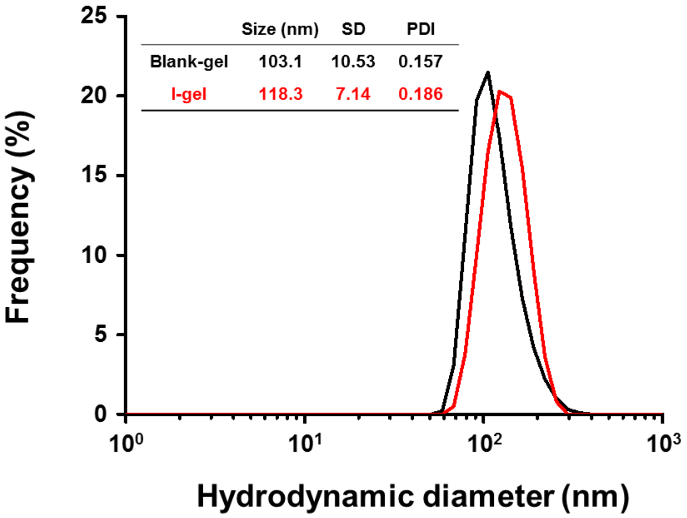

Supplementary Figure 12. Hydrodynamic size distribution of I-gel and Blank-gel. Averaged hydrodynamic diameter of (nanoscale) I-gel and Blank-gel determined by dynamic light scattering. The data show mean ± s.d., representative from 3 independent experiments.

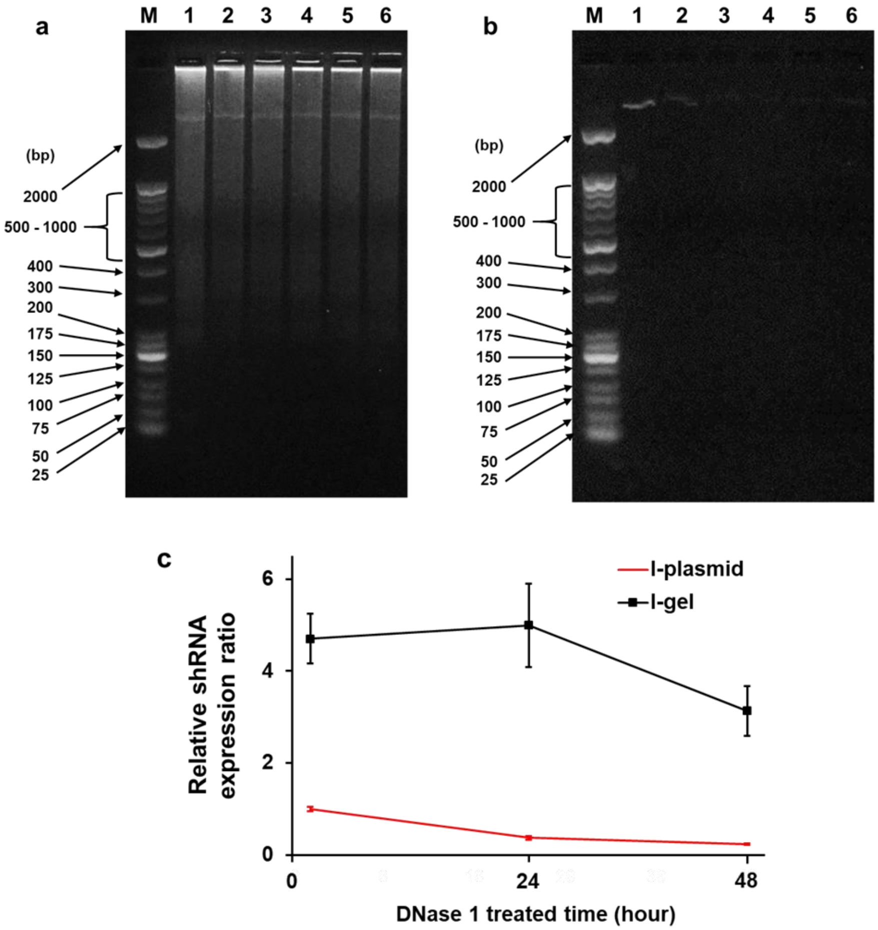

Supplementary Figure 13. Stability of I-plasmid and I-gel. Gel electrophoresis images of (a) I-gel and (b) I-plasmid treated with DNase I at 37 °C, lane 1: 0, lane 2: 1, lane 3: 4, lane 4: 12, lane 5: 24, lane 6: 48 hrs. (c) Time series of shRNA amounts transcribed from I-gel and free I-plasmid. Error bars refer to standard deviations from three replicates.

Supplementary Table 4. The shRNAs transcription rate per I-plasmid template.

DNase I treatment time	Sample	Conc. of shRNA (nM)	The number of shRNA per one plasmid template
0 h	I-plasmid	13.09 ± 0.29	18.71 ± 0.42
	I-gel	61.54 ± 3.15	87.91 ± 4.50
24 h	I-plasmid	4.82 ± 0.18	6.89 ± 0.25
	I-gel	65.39 ± 3.37	93.41 ± 4.81
48 h	I-plasmid	3.06 ± 0.05	4.37 ± 0.08
	I-gel	40.99 ± 3.38	58.55 ± 4.82

The data show mean ± s.d., representative from 3 independent experiments. The number of shRNA per plasmid template was determined by the calibration curve of shRNA concentration.

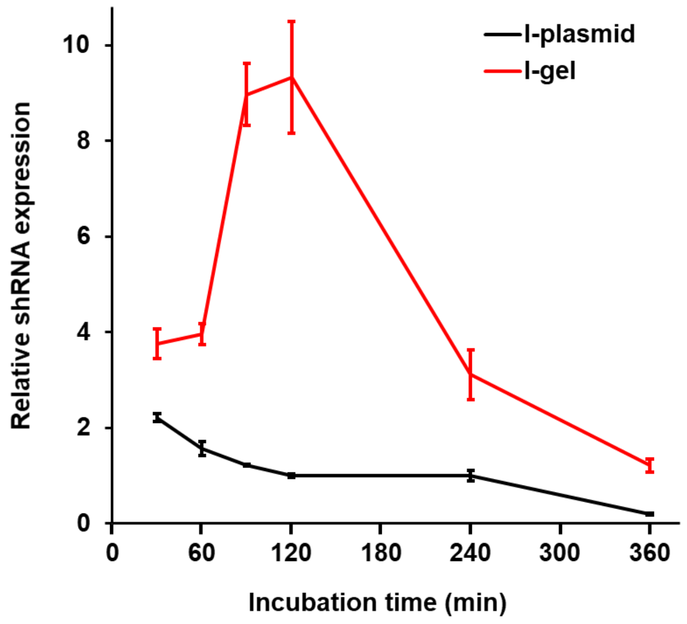

Supplementary Figure 14. Comparison of transcription efficiency of I-gel and I-plasmid. Time series of shRNA production has been monitored. Error bars refer to standard deviations from three replicates.

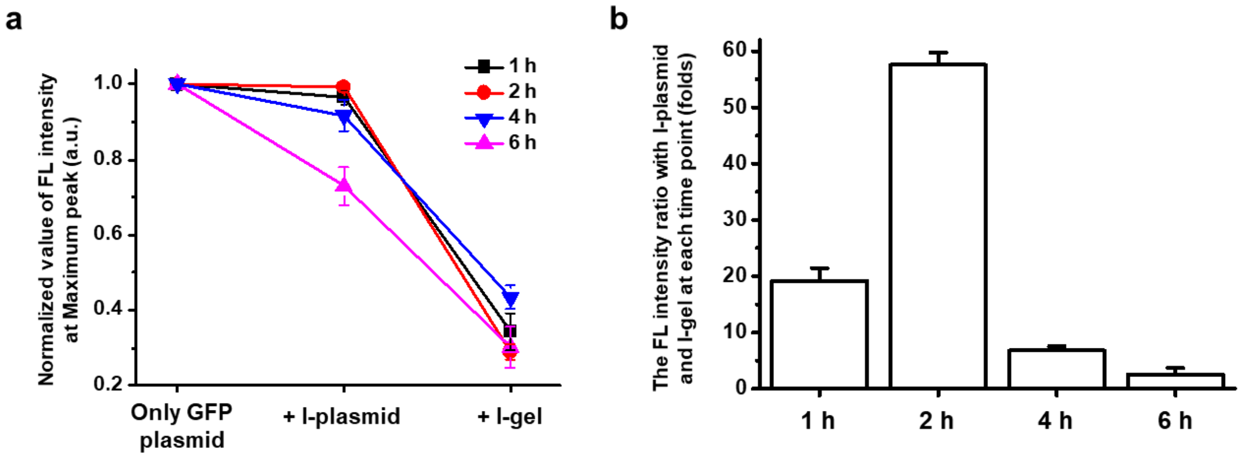

Supplementary Figure 15. The FL intensity ratio with I-plasmid and I-gel at 1, 2, 4, and 6 hours reaction time. (a) The normalized FL intensity only GFP, the addition of I-plasmid or I-gel at each reaction time. (b) The FL intensity ratio with I-plasmid and I-gel at each time points. Error bars refer to standard deviations from three replicates.

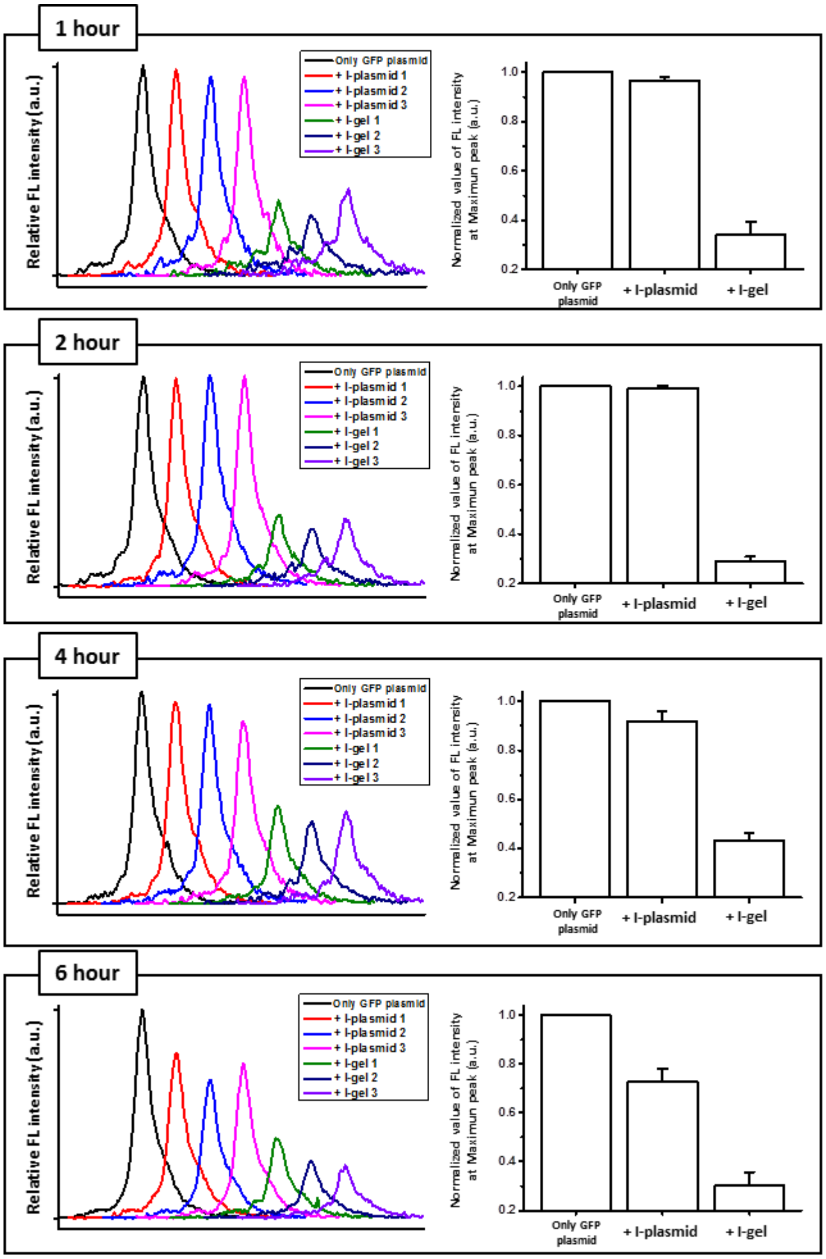

Supplementary Figure 16. The relative FL spectra of expressed GFP and normalized intensity value at various reaction time condition. The FL intensity change of GFP was measured adding I-plasmid or I-gel at 1, 2, 4, and 6 hours reaction time. Error bars refer to standard deviations from three replicates.

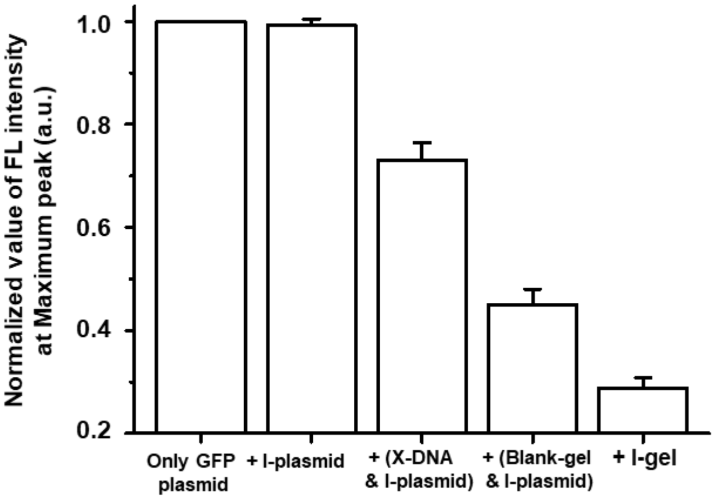

Supplementary Figure 17. The relative GFP FL intensity value of only GFP plasmid and then, the addition of I-plasmid, mixture of X-DNA and I-plasmid, mixture of Blank-gel and I-plasmid, and I-gel (variations of I-gel-forming components). Error bars refer to standard deviations from three replicates.

Supplementary Table 5. Oligonucleotide sequences of X-DNAs, inserted sequence to I-plasmid, and primer sequence for PCR. The X-DNAs and primers were used to synthesis DNA hydrogel scaffold, and find and quantify shRNA or GFP mRNA using RT-PCR or qPCR, respectively.

**X-DNA**		
	Sticky-end	Main body sequence
X01		5'- CGA CCG ATG AAT AGC GGT CAG ATC CGT ACC TAC TCG-3'
X02	5'-p-GATC	CGA GTA GGT ACG GAT CTG CGT ATT GCG AAC GAC TCG-3'
X03	5'-p-GATC	CGA GTC GTT CGC AAT ACG GCT GTA CGT ATG GTC TCG-3'
X04	5'-p-GATC	CGA GAC CAT ACG TAC AGC ACC GCT ATT CAT CGG TCG-3'
**I-plasmid (siRNA expression plasmid)** (inserted sequence)		
Spacer	Expression sequence	Spacer and Restriction site (hybridization site with X-DNA)
5'- GTG TCT TC	CG CAA GCT GAC CCT GAA GTT CAT TCA AGA GAT GAA CTT CAG GGT CAG CTT GCT TTT TT	C TCG AGG GTA CCG GAT CC-3'
**Scrambled I-plasmid (negative control RNA expression plasmid)** (inserted sequence)		
Spacer	Expression sequence	Spacer and Restriction site (hybridization site with X-DNA)
5'- GTG TCT TC	N_{52} (random base 52mer)	C TCG AGG GTA CCG GAT CC-3'
**Primer** (for RT-PCR or qPCR)		
	Forward	Reverse
shRNA	5'- CTT CAG GGT CAG CTT GCT TT-3'	5'- GCG AGC ACA GAA TTA ATA CGA C-3'
GFP mRNA	5'- GTC TTT CCC CTC TCG CCA AA-3'	5'- TTC TGC TTG TCG GCC ATG AT-3'
RT-primer	5'-GCG AGC ACA GAA TTA ATA CGA CTC ACT ATA GG(T)$_{12}$VN-3' (where V is A, C and G, and N is A, C, G and T)	

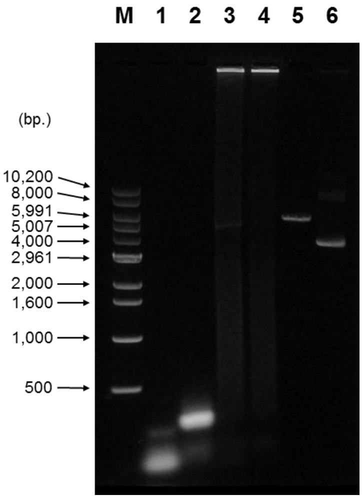

Supplementary Figure 18. Agarose gel electrophoresis image showing the DNA fragments for cell-free system. Note: DNA was resolved on 0.7% agarose gel stained with 1x Gel-red. lane M: Molecular weight standard (0.5 ~ 10.2 kbp DNA). lane 1: 36 base single strand DNA (one element composing of X-DNA); lane 2: 3 sticky-ends X-DNA; lane 3: I-gel (cross-linked with X-DNA and I-plasmid); lane 4: Blank-gel (cross-linked with X-DNA without I-plasmid); lane 5: pcDNA3.1(+) IRES GFP plasmid (GFP expression plasmid) (circular form, 6,777 bp); lane 6: I-plasmid (linear form).

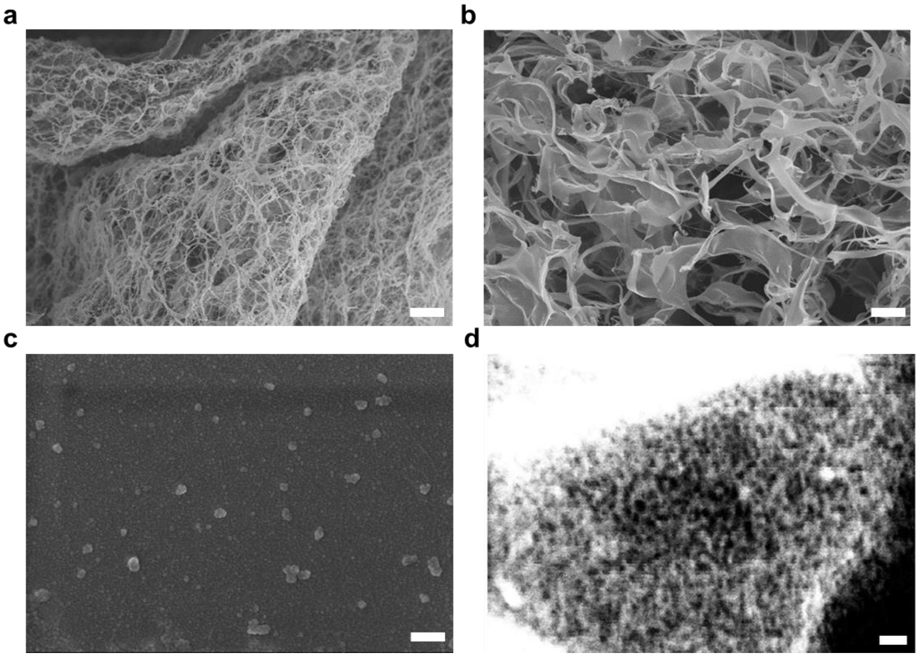

Supplementary Figure 19. SEM images of I-gel. (a, b) SEM images of large scale I-gel and (c, d) nano-scale I-gel at the ratio of 1,500:1 of X-DNA and plasmid. Scale bar: a) 20 μm, b) 10 μm, c) 100 nm, and d) 10 nm.
